# Can civil lawsuits stem the tide of direct-to-consumer marketing of unproven stem cell interventions

**DOI:** 10.1038/s41536-018-0043-6

**Published:** 2018-02-19

**Authors:** Claire Horner, Evelyn Tenenbaum, Douglas Sipp, Zubin Master

**Affiliations:** 1 Baylor College of Medicine, One Baylor Plaza, Suite 310D, Houston, TX 77030 USA; 2 Albany Law School, 80 New Scotland Avenue, Albany, NY 12208-3494 USA; 3 Alden March Bioethics Institute, Albany Medical College, 47 New Scotland Avenue, MC 153, Albany, NY 12208-3478 USA; 4 RIKEN Center for Developmental Biology, 2-2-3 Minatojima, Minamimachi Chuo-ku, Kobe, Japan; 5 Department of Physiology, Keio University School of Medicine, 35 Shinanomachi, Shinjuku-ku, Tokyo, 160-8582 Japan; 6 Keio University Global Research Institute, 2-15-45, Mita, Minato-ku, Tokyo 108-8345 Japan; 7 RIKEN Center for Advanced Intelligence Project, Nihonbashi 1-chome Mitsui Building, 15th floor, 1-4-1 Nihonbashi, Chuo-ku, Tokyo 103-0027 Japan; 8 Biomedical Ethics Research Program, Mayo Clinic, 200 First Street SW, Rochester, NY 55905 USA

## Abstract

The sale of unproven stem cell interventions (SCIs) by commercial entities has proliferated in highly developed countries, most notably in the USA. Yet, there have been few criminal prosecutions and regulatory enforcement actions against providers who have violated laws and best practice standards due to the lack of resources and legal ambiguity. While the stem cell research community has invested much in protecting patients and preventing the growth of this industry, some patients are seeking remedies under civil law to hold stem cell clinics responsible for fraudulent practices. Several patients have filed lawsuits against providers demanding compensation for physical injuries caused by unproven treatments and financial losses due to fraud and false advertising. Lawsuits can be used as a tool not only to compensate plaintiffs but also to achieve positive public health and policy outcomes. In this paper, we explore the value of a public health litigation strategy as a countermeasure against the exploitative practices of the unproven SCI industry by analyzing stem cell lawsuits and comparing them with other major public health litigation efforts. We argue that stem cell lawsuits complement other approaches to reining in unsafe practices. In particular, stem cell lawsuits could intensify publicity and raise awareness of the harms of unproven treatments, set legal precedent, reshape the media narrative from one focused on the right to try or practice to one highlighting the need for adequate safety and efficacy standards, and encourage authorities to turn their attention to policy reform and enforcement.

## Introduction

The direct-to-consumer marketing of unproven stem cell interventions (SCIs) has emerged amid a complex and highly dynamic social, economic, and legal-regulatory environment.^1^ In the early years, it was suspected that lax regulations and enforcement permitted businesses to thrive, primarily in developing countries. However, recent reports show a significant rise of clinics in economically developed countries, including the USA, UK, Australia, and Japan,^2–4^ calling into question whether robust regulatory frameworks are sufficient to rein in these practices. Although active enforcement with more stringent penalties might be an effective countermeasure, the current trend in the USA and other countries focuses on developing pathways for accelerated or conditional approval of regenerative medicine products, rather than a move toward more regulation.^5^ Indeed, most US states have enacted Right to Try legislation,^6^ and the US Senate recently passed the federal Trickett Wendler Right to Try Act,^7^ which may restrict regulatory authorities’ ability to exert control over companies selling unproven SCIs. In the absence of government oversight of private sector firms, patients and consumers may need to look elsewhere to protect their interests. Civil litigation provides a means for patients who feel they have been harmed by undergoing a SCI to seek redress and compensation from providers and may also motivate government and industry to address the issue on a larger scale.

We identified 9 individual and class action lawsuits filed by 19 named injured patients against providers offering SCIs using the LexisNexis and Westlaw databases, as well as Google.com. We excluded cases involving noninvasive cosmetic products, lawsuits that did not involve an injured plaintiff suing a clinic or clinician for damages, and cases in which the injury was not caused by the SCI. A more diverse list of cases involving stem cells that includes other claims, such as intellectual property and vendor contract disputes, has been compiled elsewhere.^8^ In this paper, we compare the identified cases to other public health litigation efforts and outline the advantages and shortcomings of civil law in protecting the health of consumers in this context. We argue that civil litigation is a potentially effective tool against businesses marketing unproven SCIs and outline how this can be achieved. We provide several considerations for members of the stem cell research community to support patients seeking legal recompense.

## A comparison with public health litigation: benefits and limits of lawsuits against providers selling unproven SCIs

The small but growing number of individual and class action lawsuits filed against stem cell clinics include claims of product liability, misrepresentation of fact, false advertising, lack of informed consent, and financial elder abuse (Table 1). The increasing frequency of lawsuits could be the beginning of a public health litigation campaign, which may serve to raise public awareness about harmful business practices and curtail the industry similar to other public health litigation strategies.

**Table 1:** Summary of US individual and class action lawsuits on unapproved SCIs

Case name	Stem cell/tissue source	Delivery of SCI	Condition(s) treated	General claims raised	Disposition
Ben Hang Lee et al. v. Human Biostar, Inc., f/k/a RNL Life Science, Inc. et al. No. 12-CV-05668 (C.D. Cal. removed June 29, 2012).	Adipose tissue from plaintiff’s abdomen	IV infusion and injection into knee	Arthritis, high blood pressure, diabetes	Misrepresentation; false advertising; negligence; financial elder abuse	Settled
Edward P. Hones v. Henry Young, et al. No. A-12-667133 (Nev. Dist. Ct. Clark Co. filed August 20, 2012).	Blood drawn from plaintiff	IV infusion	Bronchiectasis	Negligence; breach of fiduciary duty; lack of informed consent; fraud; negligent misrepresentation of fact	Settled
Tanya Enholm v. Steven R. Cohen, M.D. et al. No. 37-2013-57742-CU-MM-CTL (Ca. Super. Ct. San Diego filed July 17, 2013).	Adipose tissue from plaintiff	Injection into breast tissue	Completed as part of breast implant replacement procedure	Medical malpractice; battery; fraud; libel; product liability	Summary judgment in favor of defendants (Dismissed on appeal)
Patsy Bade v. Bioheart, Inc. et al. No. 2015-021463-CA-01 (Fla. Miami-Dade County Ct. filed September 17, 2015).	Adipose tissue from plaintiff	Injection into eyes	Macular degeneration	Product liability	Settled
Elizabeth Noble v. U.S. Stem Cell, Inc. f/k/a Bioheart, Inc. et al. No. CACE15021101 (Fla. Broward County Ct. filed November 30, 2015).	Adipose tissue from plaintiff	Injection into eyes	Macular degeneration	Product liability	Settled
Tammy Rivero v. Lung Institute, LLC, 8:17-CV-03113 (M.D. Fla. removed December 29, 2017)^a^.	Hematopoietic and/or mesenchymal stem cells from blood and/or bone marrow from plaintiff(s)	IV infusion	Lung disease (various)	Deceptive and unfair trade practices; fraudulent inducement; misrepresentation; civil penalty for criminal activity; conversion; communications fraud and misleading advertising	Ongoing^c^
Selena Moorer v. StemGenex Medical Group, Inc. et al. No. 16-CV-2816 (S.D. Cal. filed August 22, 2016)^a^.	Adipose tissue from plaintiff(s)	IV infusion and/or direct injection	Lupus;^b^ diabetes^b^	Unfair business practices; fraud; negligent misrepresentation; unjust enrichment; financial elder abuse	Ongoing^c^
Colleen Steinberg v. American Advanced Medical Corporation et al. No. BC640771 (Cal. Super. Ct. Los Angeles Co. filed November 15, 2016).	Adipose tissue from plaintiff(s)	Injection into face	Cosmetic purposes (“Stem Cell Lift”)	Medical malpractice; breach of warranty; negligent misrepresentation; intentional infliction of emotional distress; negligent infliction of emotional distress	Voluntarily dismissed by plaintiff
Jeannine Mallard v. U.S. Stem Cell, Inc. f/k/a Biohart, Inc., et al. No. CACE-17-022427 (Fla. Broward County Ct. filed December 12, 2017).	Adipose tissue from plaintiff	Injection into eyes	Macular degeneration	Product liability	Ongoing^c^

^a^Proposed class action

^b^Ailment of named plaintiff(s) only

^c^As of January 17, 2018

Public health litigation has been used in tobacco-related illness, gun violence, and the clergy abuse scandal among others, not only to compensate injured plaintiffs but also to achieve positive public health and policy outcomes.^9,10^ While the subject matter in these cases varies widely, tort litigation has had the effect of attracting public attention, shaping the media narrative, uncovering information, and giving issues greater prominence on agency agendas, eventually altering industry practices.

Public health litigation has been implemented in various ways based on the context of harm and with varying success. In the context of the clergy sexual abuse scandal, where this public health litigation strategy achieved positive outcomes, successful litigation garnered media attention, identifying sympathetic plaintiffs, generating moral outrage, and mobilizing support for victims.^11^ As the media narrative grew, more individuals came forward to file lawsuits refocusing attention on institutional responsibility, rather than solely on individual defendants. Although the primary goal of public health litigation is to compensate victims, it had the additional effect of increasing awareness and prompting governments and others, including the US Conference of Catholic Bishops, to investigate the issue and amend policies to strengthen enforcement and prosecution efforts to prevent future incidents.^11^ While lawsuits against stem cell clinics are not to target a single institution as in the clergy abuse context, several clinics do form conglomerates that might help in uncovering fraudulent business practices instituted across clinics.

The threat of financial liability for wrongdoing is the primary means by which civil law governs behavior in the private sector. Despite calls for stepping up enforcement efforts, the US Food and Drug Administration (FDA) is currently restricted in its ability to identify and target clinics operating in apparent violation of regulations.^12^ The fear of tort liability may provide sufficient incentive for compliance and minimize the occurrence of unethical practices.^9^

In addition to having a direct financial impact on clinics, stem cell lawsuits may help raise public awareness and influence public policy, particularly if there is a high volume of cases. Although public awareness and education campaigns publicize predatory practices of some stem cell clinics,^13^ the impact of these messages is being overwhelmed by clinics advertising treatments as panaceas for treatment-resistant and chronic conditions^14^ and political advocacy groups demanding easier access to unproven treatments as a matter of patient rights. Litigation may impact the broader conversation about unproven SCIs by drawing attention to negative outcomes and engendering moral outrage on behalf of vulnerable and sympathetic plaintiffs. Plaintiffs will receive public sympathy because they typically have debilitating diseases and turn to clinics as a last resort. Additionally, stem cell lawsuits may reframe the media narrative shifting the focus away from the patients’ *right to try* and instead toward misconduct by providers, holding them accountable and highlighting the need for adequate safety and efficacy testing of experimental products.

As seen with lawsuits against tobacco companies, another major benefit of stem cell lawsuits is uncovering previously undisclosed information about a provider’s practices through the discovery process, which may trigger FDA investigations.^9^ Lawsuits could help identify wrongdoers and contribute to effective enforcement against such clinics.

Finally, and perhaps most importantly, cases against providers may catch the attention of the media leading other victims to come forward and likewise file suit—similar to what happened in the clergy abuse context. Despite evidence of injury, very few patients publically report suffering negative impacts. There may be several complex reasons for patient silence; perhaps it is due to embarrassment or fear of reprisal from their patient community or perhaps they themselves do not view their overall experience as negative. However, if enough lawsuits are filed, the debate will be thrust into the public eye forcing policy makers and administrative agencies to do more to address the proliferation of clinics operating outside accepted scientific and medical standards.^9^

## A closer look at stem cell lawsuits

Most lawsuits to date have reached a settlement with no substantive ruling or court opinion on the merits of the claims raised. However, in one current proposed class action case, Moorer v. StemGenex, the court decided that some claims lack merit, while others potentially have merit and could proceed to trial.^15^ The complaint relied primarily on false advertising and misrepresentation claims, alleging that StemGenex (1) misrepresented satisfaction ratings on its website by stating that no patients were dissatisfied despite being presented with evidence to the contrary, and (2) advertised its treatment as providing a benefit without any evidence of efficacy.^15^ The Court allowed the misrepresentation of customer satisfaction claims to proceed but dismissed the claims based on a misrepresentation of efficacy. According to the Court, plaintiffs cannot prevail on a claim for fraud under California Consumer Protection Laws when there is merely a lack of evidence that a SCI will work; plaintiffs only have a viable fraud claim if they can provide positive scientific evidence both that the SCIs do not work and the clinic knew about it.^15^ The burden of proof is on the plaintiff to show that the SCIs are demonstrably ineffective and that the defendant provided them in spite of this scientific evidence. The Court also dismissed the plaintiffs’ claim that the defendants violated the human experimentation law by failing to obtain adequate informed consent for treatment. The Court found that the SCIs offered by StemGenex would not be considered “medical experimentation”, which is defined in the law as an intervention “not reasonably related to maintaining or improving the health of the subject”.^15,16^ Because the SCI was related to maintaining or improving health, it fell outside the scope of the California law.

Initial complaints often include several theories of liability, and therefore dismissal of some claims, with the option of amending and refiling them, is not necessarily a “loss” for plaintiffs. In response to the ruling, the plaintiff submitted a Fourth Amended Complaint relying on false misrepresentation of patient satisfaction ratings.^17^ This document names five plaintiffs individually in the lawsuit but proposes there are likely hundreds more plaintiffs who would be included in the lawsuit if the Court decides to certify the class. The Court’s ruling in dismissing some claims does not mean that they would not be viable in other states. As more courts issue opinions on the merits of certain claims in different jurisdictions, attorneys will be better able to identify strategies most likely to be successful in future cases.

Other lawsuits that resulted in settlement alleged different claims that may also be found to have merit. For example, in two cases against US Stem Cell, Inc. (formerly Bioheart, Inc.)^18^ and Bioheart, Inc.^19^ in 2015, plaintiffs raised product liability claims alleging that the company retrieved adipose tissue and injected it bilaterally into their respective eyes to treat macular degeneration resulting in severe and permanent damage.^20^ Unlike the StemGenex case, these cases alleged actual physical injury caused by the SCIs and claimed that the company failed to ensure both the safety of its products and that the products would successfully accomplish what they were marketed to do, namely, treat macular degeneration.^18,19^ Such cases may have a stronger basis for liability, relying on physical injury rather than financial injury and claiming that the advertised therapies are actively harmful, not merely ineffective.

However, most lawsuits filed against clinics to date have ended in settlement or voluntary dismissal with no court opinion to offer judicial analysis of the claims (Table 1). It is more difficult to gain media attention if there is no decision or the cases result in a confidential settlement. Nevertheless, a judgment against a stem cell clinic would set precedent in that jurisdiction for legal liability in offering unproven SCIs, putting other clinics on notice that similar practices may end up in a successful lawsuit. Furthermore, trial court proceedings and subsequent court opinions would be public, fostering media coverage and public policy debate. While lawsuits ending in confidential settlement may be less effective in garnering media support, an overall increase in the number of lawsuits filed may signal a public health problem in offering unproven SCIs. This is especially true because filing the lawsuit itself may result in press coverage, as has been the case for several such suits to date. Media attention would in turn heighten awareness about clinic practices and the state of the science and could raise political support to promote further regulatory inquiry into business practices.

While there is little legal precedent in the USA, one case in Japan serves as an example of a successful civil lawsuit resulting in a judgment against a stem cell clinic.^21^ In this case, the plaintiff successfully sued a private stem cell clinic for failing to discuss the risks of infusing mesenchymal stem cells, particularly in light of the patient’s preexisting medical conditions. According to the court, the plaintiff was not adequately informed of the risks prior to undergoing the procedure and was awarded the costs of therapy as well as an additional 500,000 yen (~4188 USD) in damages.^21^ Although the US administrative and legal systems differ greatly from Japan’s, this case demonstrates the global relevance of legal efforts to protect patients from unproven SCIs and offers insight into the way in which the medical community can respond.

## Combining strategies to counter the marketing of unproven SCIs

While lawsuits are likely to be effective in combating unethical stem cell clinics, the nature of the SCI industry may make it difficult for civil litigation alone to counter the current growth trend. The primarily online marketing presence of the industry and geographical mobility of providers makes it simple for providers to alter practices to evade prosecution or skip payment. While judgments could be entered against clinics, jurisdictional limitations may render successful plaintiffs unable to collect damages from defendants who relocate overseas. Therapeutic statements can be worded suggesting efficacy without making any binding promises, and the nature of web marketing allows for the rapid revision of legally problematic claims. Clinics have begun seeking ways to limit their customers’ avenues for legal recourse through the inclusion of waivers and disclaimers in informed consent documents and on websites.^22^ Non-disparagement clauses and arbitration agreements in service contracts are increasingly being used by businesses to shield reputations and limit liability. While the use of civil law as an instrument for patients seeking restitution can impact and alter business practices, having a more comprehensive approach involving litigation, federal drug and professional regulation, and public education is likely to have a collective social impact to reign in unethical practices.^13,23,24^

Regulatory efforts have been modestly successful in the closure of clinics abroad^25^ and in the USA,^26,27^ with most recent efforts by the FDA against clinics in California and Florida.^12^ Yet despite notable efforts by the FDA through investigation and drafting clearer guidance, clinics in the USA continue to offer unproven SCIs under incorrect claims of regulatory compliance^28^ or may relocate to a more permissive regulatory environment.^29^ Press coverage of lawsuits may raise awareness and draw the FDA’s attention to unethical stem cell business practices. Public health litigation may also raise the specter of unproven stem cell treatments and could result in greater allocation of resources toward FDA’s regulatory and enforcement efforts. Additionally, lawsuits can prompt state medical boards to investigate and discipline those physicians who practice beyond their scope.

Increased media exposure combined with the development of sophisticated health literacy strategies might sway some patients from turning to unproven SCIs. Patients with untreatable or refractory conditions, who may believe they have little to lose, may ignore warnings from physicians or patient advocacy groups and instead incur substantial costs and attempt unproven SCIs even with slim to no chances of improvement. While there may be a perception among some patients that clinics engaged in misconduct are simply “bad apples”, public education campaigns may gain greater traction when accompanied by media exposure and regulatory efforts. Raising awareness may motivate patients to further scrutinize clinics by demanding more information and proof of efficacy of SCIs or possibly reconsider the entire venture. Additionally, given that social support can impact self-efficacy and health behavior, broader public awareness about the dangers of unproven treatments among families and friends of patients may further influence patients’ perspectives on the risks of unproven SCIs.

Influencing the health of a population requires a combination of legal, regulatory and educational efforts.^30^ The success of anti-smoking campaigns took a combination of health litigation efforts against “big tobacco”, regulatory and policy efforts to limit smoking, and public health literacy to make a significant impact on rates of smoking.^31,32^ Combining legal, regulatory, and educational efforts offer a powerful strategy that when applied in concert could effect public policy change and stifle the burgeoning SCI industry.

## What can the stem cell research community do?

Litigation should be accompanied by coordinated efforts of scholars, patient advocacy groups, and scientists within the stem cell research community. Public outreach efforts to communicate the ethical and legal concerns to patients should continue. Efforts to raise awareness and share legal strategies with legal practitioners through bar association journals and continuing education programs will inform lawyers about harmful practices so they may better serve their clients. Scholars and stem cell scientists can provide expert testimony where appropriate regarding the state of stem cell research. Finally, patient advocacy groups should continue to provide education and outreach to reinforce the message that many SCI businesses offer scientifically baseless interventions that are unlikely to help patients or society.^33,34^

## Conclusion

A sufficient number of stem cell lawsuits can have a strong impact in stifling the unproven SCI industry by permitting patients to recover damages incurred, raising public awareness and political support, and stopping harmful business practices. While successful cases leading to judgment for plaintiffs will be most effective, settled lawsuits may also garner attention by demonstrating the need for policy changes and greater FDA oversight. As lawsuits increase, more victims are likely to join the growing chorus of advocates pushing to stop the spread of unapproved SCIs. While patients may believe they have *nothing to lose* in trying an unproven SCI, there is a real risk of losses to the patients themselves, to others suffering from intractable medical conditions, and to society. Civil litigation efforts, in conjunction with increased regulatory enforcement and public education campaigns, may convincingly show patients and society that there are real and significant harms from unproven SCIs, and this strategy may complement the arsenal of efforts focused on reining in this industry.
